# Automated Breast Ultrasound for Evaluating Response to Neoadjuvant Therapy: A Comparison with Magnetic Resonance Imaging

**DOI:** 10.3390/jpm14090930

**Published:** 2024-08-31

**Authors:** Michele Telegrafo, Stefania Luigia Stucci, Angela Gurrado, Claudia Catacchio, Federico Cofone, Michele Maruccia, Amato Antonio Stabile Ianora, Marco Moschetta

**Affiliations:** 1 Breast Care Unit, University Hospital Consortium Policlinico of Bari, Piazza Giulio Cesare 11, 70124 Bari, Italy; michele.telegrafo@policlinico.ba.it (M.T.); luigiastefania.stucci@policlinico.ba.it (S.L.S.); 2 Department of Precision and Regenerative Medicine and Ionian Area (DiMePRe-J), Aldo Moro University of Bari Medical School, Piazza Giulio Cesare 11, 70124 Bari, Italy; angela.gurrado@uniba.it (A.G.); michele.maruccia@uniba.it (M.M.); 3 DIM—Interdisciplinary Department of Medicine, Section of Diagnostic Imaging and Radiation Oncology, Aldo Moro University of Bari Medical School, Piazza Giulio Cesare 11, 70124 Bari, Italy; claudia.catacchio@uniba.it (C.C.); f.cofone@studenti.uniba.it (F.C.); amatoantonio.stabileianora@uniba.it (A.A.S.I.)

**Keywords:** breast cancer, automated breast ultrasound (ABUS), magnetic resonance imaging (MRI), neoadjuvant chemotherapy (NAC)

## Abstract

**Background:** Neoadjuvant chemotherapy (NAC) is currently used for treating breast cancer in selected cases. Our study aims to evaluate the role of automated breast ultrasound (ABUS) in the assessment of response to NAC and compare the ABUS results with MRI. **Methods:** A total of 52 consecutive patients were included in this study. ABUS and MRI sensitivity (SE), specificity (SP), diagnostic accuracy (DA), positive predictive value (PPV), and negative predictive value (NPV) were calculated and represented using Area Under ROC Curve (ROC) analysis, searching for any significant difference (*p* < 0.05). The McNemar test was used searching for any significant difference in terms of sensitivity by comparing the ABUS and MRI results. The inter-observer agreement between the readers in evaluating the response to NAC for both MRI and ABUS was calculated using Cohen’s kappa k coefficient. **Results:** A total of 35 cases of complete response and 17 cases of persistent disease were found. MRI showed SE, SP, DA, PPV, and NPV values of 100%, 88%, 92%, 81%, and 100%, respectively, with an AUC value of 0.943 (*p* < 0.0001). ABUS showed SE, SP, DA, PPV, and NPV values of 88%, 94%, 92%, 89%, and 94%, respectively, with an AUC of 0.913 (*p* < 0.0001). The McNemar test revealed no significant difference (*p* = 0.1250). The inter-observer agreement between the two readers in evaluating the response to NAC for MRI and ABUS was, respectively, 0.88 and 0.89. **Conclusions:** Automatic breast ultrasound represents a new accurate, tri-dimensional and operator-independent tool for evaluating patients referred to NAC.

## 1. Introduction

Breast cancer affects millions of women each year but advancements in medical technology and treatment approaches have significantly improved survival rates and outcomes for breast cancer patients. In the field of the medical treatment of breast cancer, neoadjuvant chemotherapy (NAC) is currently used for treating locally advanced and inoperable breast cancer and aims to reduce the tumor size and improve the survival rate, allowing for less extensive surgical procedures, better esthetic outcomes, and fewer postoperative complications [1]. Additionally, NAC allows for the early and in vivo assessment of systemic therapeutic efficacy.

Imaging tools are crucial for evaluating the loco-regional staging of breast cancer before NAC and assessing the response to NAC during and after treatment. Moreover, imaging plays an important role for identifying non-responders to NAC, allowing for the timely redirection to alternative systemic therapies and evaluating residual disease at the end of chemotherapy [2,3,4,5].

The current imaging techniques for assessing breast tumor response to NAC include digital mammography, contrast-enhanced mammography (CEM), ultrasound (both 2D and 3D), magnetic resonance imaging (MRI), and 18F-fluorodeoxyglucose positron emission tomography/computed tomography (FDG-PET/CT) [6]. In particular, MRI is considered the most accurate imaging modality for evaluating the response to NAC according to the EUSOMA guidelines [7,8,9].

However, the use of MRI has some disadvantages, including the high cost, the long acquisition time, the availability, and the need for gadolinium intravenous injection, and some general or specific contraindications can occur in some cases; furthermore, it has also been demonstrated that MRI is less accurate for assessing the tumor size of luminal subtype tumors and more accurate for triple-negative and HER2-positive tumors, and some cases of under- or overestimation have still been identified using MRI [5,6,7,8,9].

In this context, among all other imaging modalities used for evaluating the response to NAC in breast cancer, the automated breast ultrasound system (ABUS) has recently been considered for its potential role in this field [6,10,11]. In fact, ABUS provides several advantages compared to MRI, including cost-effectiveness, fast and easy use, and no need for the intravenous injection of contrast agents. Moreover, ABUS provides multiplanar high-resolution images that can be useful for surgical planning [10,11].

ABUS also shows some interesting advantages compared to handheld ultrasound (HHUS) due to being automated, tri-dimensional, and less time-consuming and operator-dependent. In fact, ABUS advanced automated algorithms allow one to provide remarkable image quality and reproducibility from user to user, with enhanced anatomic detail and remarkable time savings compared to HHUS [1,6,10].

All previous studies evaluated the role of ABUS with high-frequency linear transducers or compared the ABUS results with other breast imaging tools for the response to NAC evaluation [1,6,10,11,12,13,14,15,16,17,18,19,20,21]. In a recent study, the results of ABUS performed with a high-frequency curve transducer and contrast-enhanced ultrasound were compared for the same purpose [21].

Our study aims to evaluate the role of ABUS performed with a high-frequency (6–15 MHz) concave transducer in the assessment of response to NAC in patients with breast cancer and directly compare the ABUS results with MRI data, searching for a potential clinical role of ABUS as a valid alternative to MRI in this context.

## 2. Materials and Methods

### 2.1. Patient Selection and Inclusion Criteria

In the period between November 2022 and December 2023, a total of 52 consecutive patients (average age 47 years; range 39–58) referred to our Breast Care Unit for NAC were included in this study, all affected by nonspecial-type breast carcinomas (triple-negative breast cancers, *n* = 29; HER2+, *n* = 16; and Luminal B, *n* = 7). Each patient had undergone both MRI and ABUS examinations on the same day, before and after NAC.

### 2.2. MR Protocol

MR examinations were performed using a 1.5T system (Achieva, Philips Medical System, Best, The Netherlands) with an 16-channel phased-array breast coil. In each patient, paramagnetic gadolinium-based contrast agent with a concentration of 0.5 M was injected, using standard doses of 0.2 mL/kg of the body weight, with a flow rate of 3 mL/s. This was followed by the administration of 25 mL of saline solution at a flow rate of 3 mL/s.

The MRI protocol included the following:

- SURVEY localization sequences (axial, sagittal, and coronal) weighted in T1;
- axial T2-weighted short tau inversion recovery (STIR) turbo spin-echo (TSE) sequences, repetition time/echo time/time interval (TR/TE/TI) = 3800/60/165 ms, field of view (FOV) = 250 × 450 mm (AP × RL), matrix 168 × 300, 50 slices with 3 mm slice thickness and no gaps, 3 averages, turbo factor 23, resulting in a voxel size of 1.5 × 1.5 × 3.0 mm^3^;
- axial T2-weighted TSE sequences, TR/TE = 6300/130 ms, FOV = 250 × 450 mm (AP × RL), matrix 336 × 600, 50 slices with 3 mm slice thickness and no gaps, 3 averages, turbo factor 59, SENSE factor 1.7, resulting in a voxel size of 0.75 × 0.75 × 3.0 mm^3^;
- T1 high-resolution isotropic volume examination (THRIVE), dynamic 3D acquisitions with contrast enhancement, gradient echo, TRE/TE = 4.4/2.0 ms, FOV = 250 × 450 × 150 mm (AP × RL × FH), matrix 168 × 300, 100 slices with 1.5 mm slice thickness, turbo factor 50, SENSE factor 1.6, 6 dynamic acquisitions, resulting in 1.5 mm^3^ isotropic voxels, a dynamic data acquisition time of 1 min 30 s, and a total sequence duration of 9 min;
- diffusion-weighted imaging with background signal suppression (DWIB) sequences, TR/TE = 4600/66 ms, FOV = 300 × 338 × 145 mm (AP × RL × FH), matrix 168 × 300, b-value 2 (s/mm^2^) max b-value 700–1000 (s/mm^2^), 50 slices with 3 mm slice thickness, resulting in a voxel size of 1.34 × 1.33 × 3.0 mm^3^ (AP × RL × FH), with ADC mapping.

### 2.3. ABUS Technique

ABUS examination was performed using an Invenia 2.0 GE Healthcare device (GE Healthcare Systems, Chalfont Saint Giles, UK) with a concave wideband high-frequency line-array probe (6–15 MHz; field of view = 15.4 × 17.0 cm; imaging depth = 5 cm; reconstructed coronal slice thickness from the skin to the chest wall = 2 mm; and element pitch 0.20 mm). The patient was positioned supine with the arms above the head, and a hypoallergenic gel based on water was applied to the entire breast. All images were acquired by a trained radiologist with 5 years of experience. The transducer automatically created uniform compression across the entire breast tissue to the chest wall and was automatically moved from the superior border to the lower border of the breast to acquire the images. Markers were positioned on the nipple on the reconstructed coronal plane. For each breast, three acquisitions were obtained: anterior–posterior with the nipple at the center, lateral including the supero-external part of the breast tissue, and medial including the infero-internal part of the gland. In large-breasted women, two additional acquisitions (superior and inferior) were obtained. The acquired images were then transferred to the dedicated workstation, Invenia Viewer, and reconstructed for image analysis. A multi-slice B-mode image acquisition with frame-by-frame 3D position registration was used. In all cases, tissue equalization algorithm, nipple shadow compensation, breast border, and chest wall detection were obtained. Three-view US images were assessed with a synchronized view of multiple acquisitions in order to evaluate and cross-reference areas of interest from multiple angles. An automatic comparison tool was used in order to easily compare all regions of interest to prior exams.

### 2.4. Image Analysis

Two radiologists with 10 years of experience in breast MR imaging and 5 years of experience in ABUS imaging analyzed the ABUS and MRI images before and after NAC, describing the local extent of cancer before therapy and searching for signs of complete or partial response to therapy. The obtained results were compared with the MRI images. 

The assessment of response to therapy was primarily based on the measurement of the maximum longitudinal and transverse diameters of the tumor lesion before and, if still present, after therapy according to the Revised RECIST guidelines (version 1.1) [22]. Only the hypoechoic component of the lesion was considered in the size evaluation to ensure measurement objectivity, without considering the variable extension of the surrounding hyper-echoic component.

The ABUS and MRI findings were also compared with the histological examination after surgical treatment. In the samples collected following surgery, the tumor size (major diameter), histological type (histotype), the presence of hormone receptor positivity, and the markers of the tumor lesions were assessed.

A pathological complete response (pCR) was defined as the complete disappearance of invasive carcinoma associated with axillary lymph node negativity (ypT0 ypN0) [12]. In cases of an incomplete pathological response, the maximum diameter of the residual lesion was considered.

### 2.5. Statistical Analysis

ABUS and MRI sensitivity (SE), specificity (SP), diagnostic accuracy (DA), positive predictive value (PPV), and negative predictive value (NPV) were calculated and represented using Area Under ROC Curve (ROC) analysis, searching for any significant difference (*p* < 0.05). The McNemar test was used to search for any significant difference in terms of sensitivity by comparing the ABUS and MRI results.

The inter-observer agreement between the two radiologists in evaluating the index tumor size and extent and response to NAC for both MRI and ABUS was calculated using Cohen’s kappa k coefficient, rated as follows: k values less than 0.20 = poor, 0.21–0.40 = fair, 0.41–0.60 = moderate, 0.61–0.80 = substantial, and 0.81–1 = near-perfect agreement. 

## 3. Results

**Figure 1:**
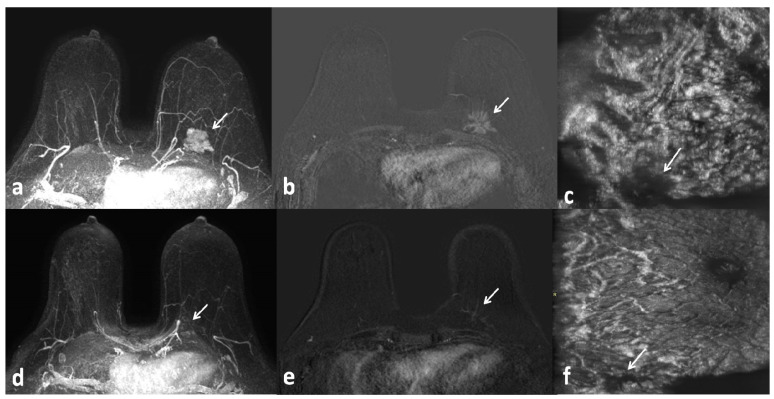
Axial MRI with maximum intensity projection (MIP) reconstructions (**a**,**d**), subtracted (post-contrast–pre-contrast) images (**b**,**e**), and ABUS coronal images (**c**,**f**) taken before (**a**–**c**) and after (**d**–**f**) NAC. Triple-negative cancer of the left breast with partial response to NAC (arrows).

**Figure 2:**
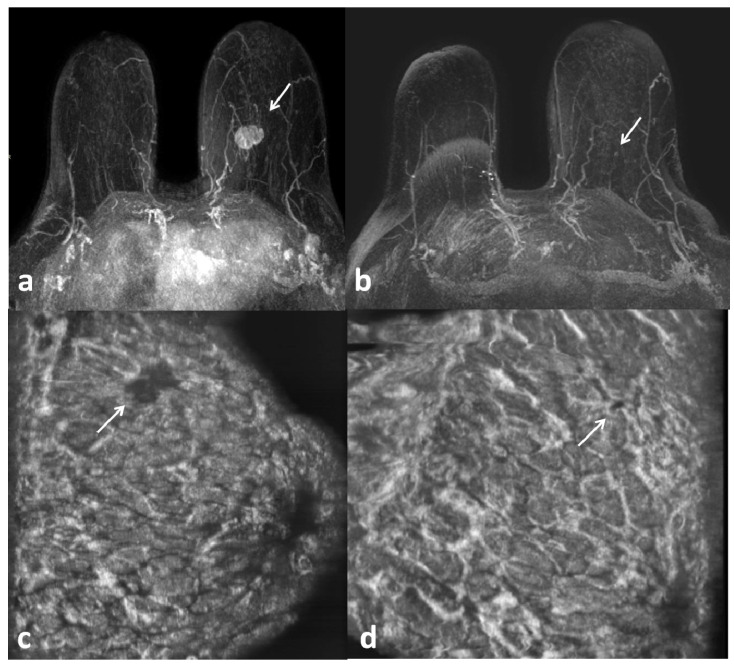
Axial MRI with maximum intensity projection (MIP) reconstructions (**a**,**b**) and ABUS coronal images (**c**,**d**) taken before (**a**,**c**) and after (**b**,**d**) NAC. HER2+ breast cancer of the left breast with complete response to NAC (arrows).

Post-NAC MRI detected 31 cases of CR and 21 cases of persistent disease, while ABUS identified 33 cases of pCR and 19 cases of persistent disease (Figure 1 and Figure 2). The mean residual tumor size was 18 mm and 19 mm, respectively, with ABUS and MRI (standard deviation of 14.62 and 17.85, respectively). The mean acquisition time was 19 ± 2 min for MRI and 9 ± 1 min for ABUS, while the mean post-processing and interpretation time was 7 ± 1 min for MRI and 3 ± 1 min for ABUS. 

After completing NAC, 42 patients (81%) underwent breast-conserving surgery and 10 patients (19%) received a mastectomy. The final histological examination of the 52 patients revealed 35 cases of pCR and 17 cases of partial response or persistent disease. 

Upon pathologic examination after surgery, the mean residual tumor size was 24 mm (range of 0–38 mm). The analysis of the obtained data revealed that both ABUS and MRI were effective in assessing the response to therapy. 

**Figure 3:**
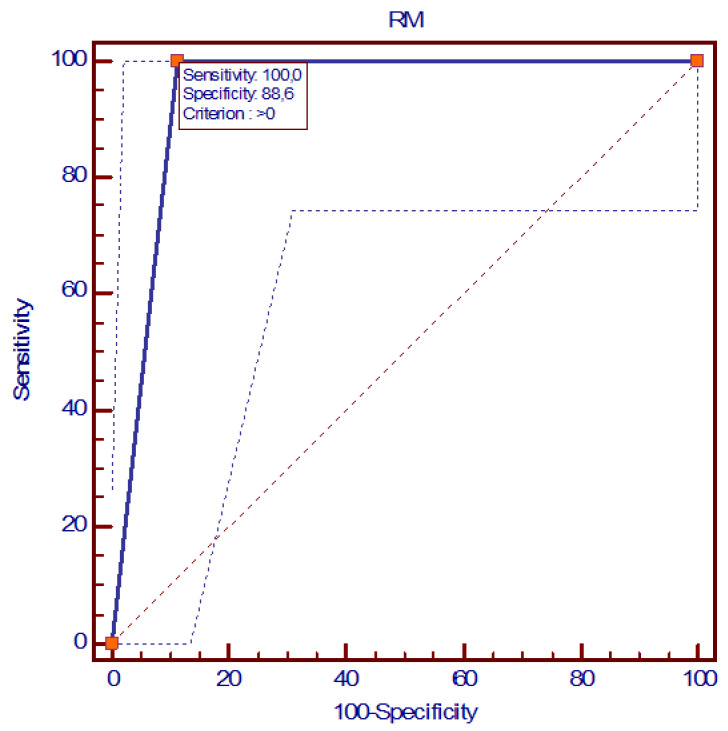
ROC curve of MRI diagnostic performance.

When compared to the histological data, in post-NAC MRI, four false positives occurred. MRI showed SE, SP, DA, PPV, and NPV values of 100%, 88%, 92%, 81%, and 100%, respectively, with an AUC (Area Under ROC Curve) value of 0.943 (95% confidence intervals: 0.841–0.988), with a *p* < 0.0001 significance level compared to HE (Figure 3).

**Figure 4:**
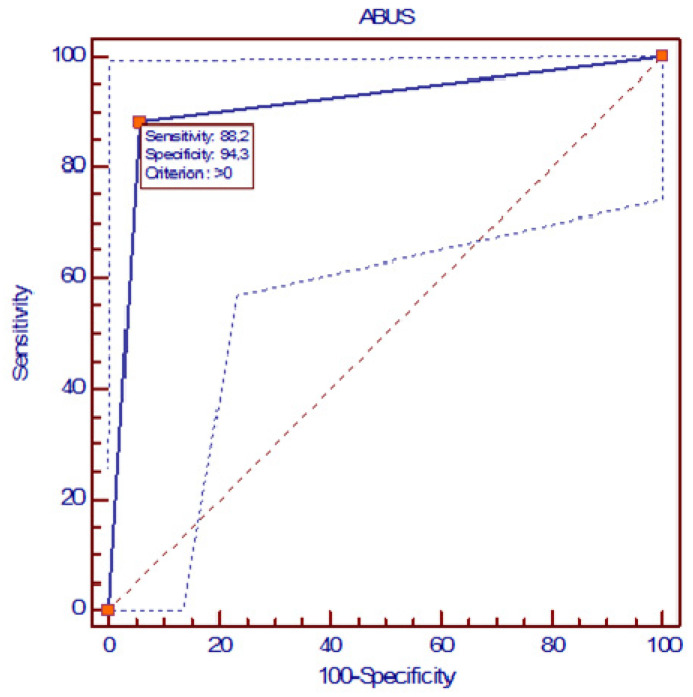
ROC curve of ABUS diagnostic performance.

In post-NAC ABUS, two false negatives and two false positives occurred. The SE, SP, DA, PPV, and NPV values for ABUS were 88%, 94%, 92%, 89%, and 94%, respectively. ABUS obtained an AUC of 0.913 (95% confidence intervals: 0.801–0.973), with a *p* < 0.0001 significance level compared to HE (Figure 4). The McNemar test compared ABUS and MRI, revealing a 7.69% difference, with a *p*-value of 0.1250, indicating that the difference between the two imaging techniques was not statistically significant (Table 1).

The inter-observer agreement between the two radiologists in evaluating index tumor size, extent, and response to NAC for MRI and ABUS was, respectively, 0.88 (95% confidence intervals = 0.821 ÷ 1.000) and 0.89 (95% confidence intervals = 0.816 ÷ 1.000).

## 4. Discussion

NAC plays a crucial role in breast cancer treatment by reducing tumor cells and significantly increasing the rate of breast-conserving surgery [13]. Histological examination currently serves as the gold standard for assessing NAC response with high diagnostic accuracy. However, NAC efficacy must be determined not only postoperatively but also during the course of chemotherapy.

MRI is currently the reference examination, playing a crucial role in disease staging, assessing the primary lesion size, local–regional disease extension, multi-focality, multicentricity, and therapeutic response analysis. Indeed, MRI provides an accurate estimate of NAC response by reflecting tissue changes based on contrast enhancement distribution, while DWI sequences offer information about tumor cellularity and membrane integrity, sensitive to intra-tumoral changes induced by chemotherapy [9].

However, MRI accuracy is affected by several factors that could alter the ability to establish residual disease with certainty, varying by the different histological subgroups. The accuracy of residual tumor evaluation varies, as Pasquero et al. [14] demonstrated. MRI is superior in the diagnosis and monitoring of HER2+ and triple-negative breast cancer compared to Estrogen+ or HER2- tumors. The chemotherapy regimen also affects the accuracy and sensitivity in predicting pCR and quantifying residual disease, with lower accuracy in patients treated with Taxanes [15].

Our study indicates that MRI has a high sensitivity (100%) but a variable specificity and a certain rate of false positives. 

In contrast, the role of ABUS in evaluating pCR after NAC is not yet fully understood. Our study demonstrates that, in terms of sensitivity and specificity, this imaging modality is similar to MRI, as previously shown by Van Egdom et al. [16]. Indeed, ABUS allows for the identification of pre-NAC architectural distortion, providing precise information about its location and initial size, and comparing the results with post-NAC findings, facilitating the planning of subsequent surgical treatments. ABUS is effective in detecting monofocal lesions, particularly for certain tumor histotypes like Luminal A and triple-negative cancers. The study of Park et al. [17] demonstrated that, among all molecular subtypes, only TNBC showed a very high correlation between imaging and histological examination when evaluated with ABUS. Furthermore, the wider probe used in this technique enables the better visualization of larger lesion, especially those that exceed the field of view (FOV) of the traditional handheld ultrasound (HHUS) probe. In fact, the wide field-of-view high-frequency transducer automatically creates uniform compression across the entire breast for consistent, reproducible image quality independent of the operator, while the convergent scan line geometry allows to minimize beam refraction [10,11].

However, ABUS has some important limitations and may not be sufficiently sensitive in differentiating therapy-induced fibrosis from hypoechoic residual tumor. Additionally, tumor fragmentation and stromal retraction make interpretation challenging, increasing the likelihood of false positives.

The importance of the complete coverage of the breast has been widely emphasized. The peripheral localization of the lesions can sometimes reduce the diagnostic performance of ABUS compared to HHUS, especially in the case of large breasts, potentially leading to missed cancer diagnoses. Furthermore, the evaluation of the axillary region is excluded from the FOV, preventing the in-depth examination of the axillary lymph nodes. 

In contrast to ABUS, HHUS examination offers the advantage of incorporating Doppler and elastography analysis. These features facilitate a differential diagnosis between benign and malignant lesions, providing not only morphological information but also crucial qualitative insights into the vascularity and elasticity of breast tissue and lesions. Equally important is the capability of HHUS to perform ultrasound-guided procedures, such as biopsies on suspicious lesions, for subsequent histological characterization by a pathologist.

Another noteworthy aspect is that, as confirmed by multiple studies, the lesion’s average diameter plays a crucial role in ABUS’s ability to detect lesions, especially as the size increases. However, this limitation is shared with HHUS. For instance, Berg et al. [18] reported a 12% increase in the ability to detect lesions per millimeter as the diameter increased from 3 mm to approximately 13 mm (with a decreasing rate of 44% for lesions sized 3.1–5 mm and a rising rate of 97% for those >11 mm). Only Wang et al. [19] demonstrated a higher diagnostic accuracy for ABUS compared to HHUS for lesions smaller than 1 cm. In our study, an almost perfect inter-observer agreement was obtained for both MRI and ABUS, demonstrating that ABUS imaging can be proposed in the field of imaging evaluation of response to NAC and that ABUS is probably less operator-dependent compared to HHUS, as reported in other recent studies [20,21].

Our study has some important limitations. First, we enrolled a limited number of patients. Second, we did not consider the different molecular subtypes and chemotherapy regimens. Third, axillary lymph node evaluation was excluded from this analysis because not anatomically included in the FOV of the ABUS technique.

## 5. Conclusions

Automatic breast ultrasound represents a new accurate, tri-dimensional, and operator-independent tool in the field of breast imaging and can be consistently proposed for evaluating patients referred to NAC in order to assess the response to therapy with regard to tumor size. Further studies on larger series are needed in order to confirm these data.

## Figures and Tables

**Table 1:** Comparison of MRI and ABUS results in evaluating response to NAC in breast cancer. No significant difference was found (*p* = 0.1250).

IMAGING TOOL	ABUS		*n* = 52
MRI	Complete Response	Persistent Disease	
Complete Response to NAC	31	0	31 (60%)
Persistent Disease	4	17	21 (40%)
*n* = 52	35 (67%)	17 (33%)	*p* = 0.1250

## Data Availability

Interdisciplinary Department of Medicine, Section of Radiology and Radiation Oncology, University of Bari “Aldo Moro”, Bari, Italy.
